# Evolving Marine Biomimetics for Regenerative Dentistry

**DOI:** 10.3390/md12052877

**Published:** 2014-05-13

**Authors:** David W. Green, Wing-Fu Lai, Han-Sung Jung

**Affiliations:** 1 Oral Biosciences, Faculty of Dentistry, The University of Hong Kong, Hong Kong, China; E-Mail: dwgreen@hku.hk; 2 Division in Anatomy and Developmental Biology, Department of Oral Biology, Oral Science Research Center, BK21 PLUS Project, Yonsei University College of Dentistry, Seoul 120-752, Korea; E-Mail: rori0610@graduate.hku.hk

**Keywords:** regenerative dentistry, biomimetics, marine invertebrates, bone, dentine

## Abstract

New products that help make human tissue and organ regeneration more effective are in high demand and include materials, structures and substrates that drive cell-to-tissue transformations, orchestrate anatomical assembly and tissue integration with biology. Marine organisms are exemplary bioresources that have extensive possibilities in supporting and facilitating development of human tissue substitutes. Such organisms represent a deep and diverse reserve of materials, substrates and structures that can facilitate tissue reconstruction within lab-based cultures. The reason is that they possess sophisticated structures, architectures and biomaterial designs that are still difficult to replicate using synthetic processes, so far. These products offer tantalizing pre-made options that are versatile, adaptable and have many functions for current tissue engineers seeking fresh solutions to the deficiencies in existing dental biomaterials, which lack the intrinsic elements of biofunctioning, structural and mechanical design to regenerate anatomically correct dental tissues both in the culture dish and *in vivo*.

## 1. Introduction

Biomaterials are widespread in clinical and experimental medicine. Synthetic and biological origins made from polymers or ion complexes, nanoparticles, quantum dots and intricate composites have been designed and fabricated for roles in tissue replacement, biomonitoring and therapeutic delivery of drugs [1]. The more that is understood about the molecular interplay, gene, cellular, biochemical and metabolic regulatory networks in health, disease, inflammation and trauma so the design of biomaterials to match and participate in recovery, regeneration and disease cures has increased in biological complexity. Now there are more sophisticated biomaterials, built from miniatures that are enriched with bioactive elements such as extracellular matrix proteins [2]. There remain startling imperfections in all biomaterials particularly regarding their responsiveness to biology, mechanical design and functional match with normal healthy tissues. Structures made with biomaterials miss important features of design such as, exquisite interfaces, proper integration of different materials and substrates biomimetic surfaces [3,4,5]. This is really crucial in the dental field, in which individual tissue units are made up of multiple complexes of tissue types. Take for example the periodontium composed as it is of sandwiched cementum, periodontal ligament tissue and bone.

Almost every type of biomaterial has been used in clinical or experimental dentistry. Metals and ceramics have been predominant in fixing and replacing the major hard tissues of the tooth. Polymers have also played a major part in dental restorations and replacements [6]. In addition, new roles are being designed for these materials. For example, the versatility that polymers offer has engendered uses in therapeutic delivery such as nucleic acids-SiRNA [7,8] and microRNA’s [9]. New categories of biomaterials are coming to the fore: self-assembled, nanofibrous, nanoparticulate that will eventually provide natural levels of biomimetic sophistication. However, they have had limited impact in dentistry for now due to lower than needed production volumes and technically difficult syntheses. Marine biomimetic structures offer useful advantages before the future onset of truly biomimetic artificial designs. The main advantage is their sophisticated pre-design of structure and architecture.

### 1.1. Striving for de Novo Regeneration of Whole Living Tooth Tissues

In dentistry, structural biomaterials that enable interactions between epithelial and mesenchymal stem cells have been used to facilitate tooth regeneration [10]. These biomaterials are typically artificial. In reconstructive maxillofacial and periodontal surgery, autologous tissues and autologous blood plasma proteins are introduced into surgery as well to potentiate regeneration and promote rapid healing and sealing [11,12,13]. For example, calcium phosphate bone substitutes and collagen derivatives are routinely used as structural frameworks that induce tissue rebuilding inside alveolar bone voids and replace missing gum tissue, respectively. These interventions can usually guarantee a clinically acceptable level of anchorage and sealant for metal teeth and implants [14]. The addition of bone morphogenic proteins (BMP’s) and Fibroblast growth factor (FGF), dental matrix proteins, induced Pluripotent Stem (iPS) cells, Periodontal ligament stem cells (PDLSC) and cell sheets into the diseased periodontal ligament have led to accurate regeneration.

Recently, the technical feasibility of using scaffolds to facilitate the regeneration of dental structure has been further evidenced by a study that transplanted stem cells with macroporous biphasic calcium phosphate (MBCP) into the dorsal subcutaneous pockets of 5-week-old male immunocompromised mice [15]. The stem cells included cells isolated from the periodontal ligament (PDL) of permanent teeth (pPDLSCs), stem cells from the PDL of deciduous teeth (dPDLSCs), and human bone marrow-derived mesenchymal stem cells (BMMSCs). All three types of stem cells could successfully produce hard tissues at the periphery of the MBCP eight weeks after the transplantation of the scaffolds [15]. The BMMSC transplants produced bone-like tissues that had a lining of osteoblast-like cells, a lamellar pattern of fiber alignment, and an outer dense fibrous tissue parallel to those osteoblast-like cells. Furthermore, embedded cells were identified to be present in the matrix, which exhibited condensed nuclei-like osteocytes and reduced cytoplasmic space in the lacunae [15]. Additionally, cementum-like tissues were formed in the pPDLSC and dPDLSC transplants [15]. Cementocyte-like cells resembling cellular cementum were also observed in some of the tissues formed. By quantitative RT-PCR and immunohistochemical analyses, higher expression levels of genes related to mineralization (BSP, osteocalcin, and osteopontin) were observed in the BMMSC transplants, whereas in the pPDLSC and dPDLSC transplants, higher expression of genes related to the cementum/PDL complex (CP23 and collagen XII) was observed [15]. These findings act as a proof-of-concept to illustrate that tooth tissue engineering in scaffolds is practically applicable. Despite this premise, autologous patient living tissue is ultimately what works best to fasten the biomaterial scaffolds securely for tooth regeneration but is generally in short supply. As a consequence, there must be a viable clinically acceptable alternative. Thus, high-quality dental tissues are being grown in tightly manufactured conditions that can be precisely controlled to match the extent of damage needing replacement.

Vital living dental tissue can be grown to a limited extent in petri dishes from donor cells and patient cells extricated from the apical papilla (SCAP), exfoliated deciduous teeth (SHED), periodontal ligament (PDLSC), pulp tissue (DPSC), and dental follicle (DFSC). A more unresolved problem has been in acquiring stem cells for recreating a dental epithelium [16]. Stem cells can be obtained from even non-dental cells and be made to grow in the position of the damaged tooth; odontogenesis in non-dental cells has been induced when exogenous BMP4 was given to intact cultures of second pharyngeal arches [17]. Though no hard tissues (such as enamel and dentine) or identifiable teeth have been obtained, organized cell layers were successfully formed in treated cells derived from the second arch cultures [17]. Further, these cells, which had been treated with BMP4 differentially, expressed *Dspp* and *amelogenin* in adjacent layers in a relative orientation similar to that in teeth [17]. These observations confirm the possibility of inducing formation of a complete tooth primordium from non-dental cells by directly manipulating intracellular levels of signaling molecules. Apart from that, tooth primordia have also been successfully procured by tissue recombination. Earlier studies demonstrated that tooth formation can be induced in aboral first-arch mesenchyme and second-arch mesenchyme by applying early oral epithelium (E9-E11) [18,19], which has also been reported to induce tooth formation in the cranial neural crest and trunk neural crest if recombined prior to migration [20]. In a more recent study, the oral epithelium of murine embryos between E9 and E11.5 was also shown to induce tooth formation in non-dental mesenchyme [21]. This accumulated evidence supports the technical possibility of regenerating tooth primordia or bioteeth for subsequent transplantation in an *ex vivo* environment. Unfortunately, replacing missing dental tissue parts with a cultivated equivalent is preferable to using man-made materials and structures engineered for the same task. It has been impossible to completely emulate the evolutionary perfected design of natural biomaterials synthetically. Conventional dental materials are therefore inclined to fail because the structural and biological properties (if they do exist) are not identically matched to their host surroundings of dentine, enamel or periodontal tissue [22]. In one large study treating 426 adults with 3 separate types of restoration up to a total of 926 median staying power of the restoration varied from 7.8 years for resins to 14.6 years for crowns [23].

Entire living tissues can be produced in the petri dish with a combination of cellular building blocks, cell and tissue supporting frameworks, and cell activating genes and protein factors in the typical tissue engineering strategy [24,25]. The archetypal petri dish has evolved and metamorphosed into quite sophisticated bioreactors, devices with microcapillaries and micron scale chips [26,27,28]. A variety of important tissues have been cultivated with a clinically acceptable degree of anatomical accuracy this way such as recently: heart tissue components, optic cup, brain cortical tissue [29,30,31,32]. In most other cases though the quantity of tissues generated exclusively in the lab can be too small-as it is restricted to 2 mm thickness for most clinical treatment to be successful [33]. This is because for technical reasons a blood vessel supply is difficult to establish in developing engineered tissues. It has been stated that almost every cell residing in the body is 200 μm from the nearest capillary in order to survive [34]. Laying down a nerve network derived from precursor cells and a conduit, inside petri-dish grown tissues is an equally vital mission to provide full and vital sensory and actuation roles to that tissue. So far synthesis of a nerve and blood vessel network-individually or in combination with the construction of any given tissue has not been achieved. Indeed, there is paucity of clinically acceptable and viable materials and structures in dentistry (to augment and replaced damaged oral and craniofacial structures) that can support and replicate tissue to a sufficient quality of function and offer credible alternatives to conventional techniques and strategies [35]. Dental tissues by nature are stacked together with each other across graded interfaces, so that to regenerate one tissue requires engineering of complete other tissues with which they are set in contact with [35]. One interesting origin for discovering usable items with the utility and versatility necessary to complete tissue generation are the products of evolution from simple animals and plants. Previously, we have demonstrated how this has yielded drug delivery devices, cell sheet templates and frameworks for cartilage regeneration [36,37,38,39]. In this article, we look at the possibilities of harnessing structures, substrates and materials from the world of marine biology for use in the tissue engineering of dental tissues, jawbone, dentine and pulp.

### 1.2. Frameworks for Tissue Engineering

Marine biomaterials are being renewed as candidates for quite broad biomedical applications. Capacities to extract sufficient marine product yields are increasing too and there is repeated evidence of effects on human cells and biology. Promising examples include collagens from jellyfish [40], polymers from marine Diatoms [41], Chitin from marine sponges [42] and hydroxyapatite and calcium phosphates from fish bone and other organisms [43]. Advantages of marine sourced biomaterials vary from inspiration to engineer materials with new properties and effects, vital biological activities and high bioavailability (Table 1).

**Table 1:** Comparison between marine biostructures and other biomaterials in regenerative medicine and including dentistry.

Marine Biostructures	Other Biomaterials and Their Structures
Pre-designed and pre-fabricated with numerous possible roles in cell and tissue regeneration. Intrinsic functions can be translated to human biology.	Fabricated by designer with many honed functions specific to biomimetic induction, control and regulation of developmental and regeneration processes.
Inherent hierarchical structural design.	Limited levels of hierarchy can be assembled, so far.
High diversity of structure and architecture. Some species highly abundant and sustainably sourced.	Structural and architectural specificity is diverse but difficult to generate artificially requiring complicated processing.
Intrinsic design features retained for fracture prevention and biorecognition (physical, biochemical, sometimes cellular).	Difficulty in generating composites with comprehensive fracture toughening.
Short processing times with low cost. Although obtaining marine biostructures from natural habitats can be expensive.	Long manufacturing process with many steps at high cost.
Some difficulties in sterility and removing all contaminants.	High levels of sterility obtained.
Safe storage indefinitely usually without special conditions.	Variable storage times with possible degradation.
Possibility of low-level immunogenicity.	Synthetics have no immunogenicity. Some natural biopolymers risk immunogenicity.
Low cytotoxicity in properly cleaned biostructures.	Low cytotoxicity.
Less utility to integrate bioactive elements.	Large range of bioactive elements (adhesive ligands, enzyme-sensitive cross-links) can be integrated biomimetically during synthesis.
Less accessible in adding new biomimetic properties.	Highly accessible design and construction with many biomimetic properties: self actuation, self-adjustment, *etc.*

## 2. Biomedical Applications of Marine Products

Bioactive compounds from marine organisms have been applied in medicine and steadily in areas of regenerative medicine. Certainly secondary metabolites from marine invertebrates and commensal microbes have been harnessed as potential drug candidates for destroying tumors, killing bacteria stopping inflammation and suppressing pain [44,45,46,47]. They are chemical rich and highly diverse although not necessarily highly abundant. However, advances in molecular biology allow prospecting for genes involved in producing bio medically significant chemical compounds, effective synthesis in bacterial vectors or synthesis from the bio molecular structure itself as well as screening methods more accommodating to natural chemical compounds [48]. Finding new niches and habitats for products is ongoing and can be fruitful pursuit for new and abundant natural marine products [44].

**Figure 1:**
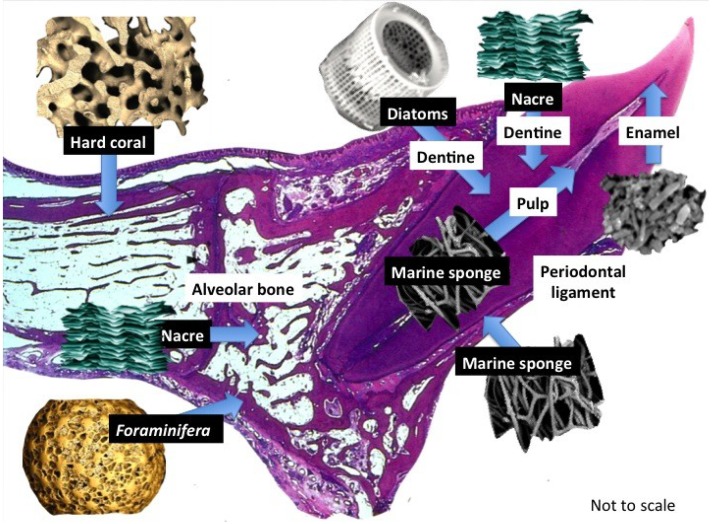
Structural products harnessed directly from marine life. In this show a small selection of marine biostructures with promising properties to rebuild the main individual tooth tissues that are affected by disease: bone, dentine, pulp and periodontal ligament. A further look into marine natural history will offer more structures destined for transplantation into the tooth structure itself. These marine derived structures are required to support dental cells and tissues in their natural position and create structural, crystalline and mineral ion clustered microenvironments for developing mineralized and connective tissues with properties that ensure seamless fusion takes place within existing tooth structures. Tissue boosting molecules such as, growth factor proteins can be stably adsorbed into the marine substitutes and also be entrapped within the mesopores and nanopores. Various routes for appropriating the structures can be employed that increase the number and type of biological roles they have. We can add more roles than the original structure to jump start regeneration.

Structures, materials and substrates extracted from the plant and animal kingdoms are products that can be implemented in most regions of the tooth in regenerative dental therapies [49,50,51,52,53,54,55]. Plant extracts are likely to provide valuable material substitutes made from polysaccharides, phytochemicals and proteins with superior cell and tissue interactivities than their synthetic counterparts [52]. However, their usage in regeneration therapies has not been fully exploited in practice because of the bias towards well-established restorative materials such as titanium [56], metal alloys [57], zirconia [58], bioactive glasses [59], calcium phosphates [60,61] and glass ionomer resins [62,63]. Many of these are now being gradually being adapted at the Nano metric scale to improve many functions and even superseded by a slowly increasing number of Nano biomaterials and biomimetic and bio inspired materials and structures but these have been limited to fabrication of coatings, new fillers, anti-bacterial factors and for tooth remineralisation strategies [63,64,65,66,67]. We are looking at exploiting those products that have previously evolved in early and simple *life-forms* exemplified by marine sponges and planktonic organisms. Ultimate biomimicry is the ability to emulate and simulate human biology [37]. In the meantime, there is a strong case for using natural products of all types in regenerative strategies requiring a framework because of their pre-organization into superior and sophisticated structures. The aim is to continue closing the gap between materials and biology and be better able to merge the two into a single product. We present data demonstrating the utility for clinical therapy of an assortment of easily obtained marine products for selected roles in regenerative dentistry-incorporating oral tissues and craniofacial tissues in augmenting and replacing missing, damaged and diseased bone, dentine and periodontal ligament tissues (Figure 1).

### 2.1. Source of Marine Biomaterials and Products

In the past, products from the living marine environment have been directly implemented in regenerating bone, cartilage and fat tissue to complete the musculoskeletal system [68]. Pre-made structures synthesized in nature have advantages over artificially made structures in a number of crucial ways. Pre-made structures are hierarchical, exhibit intricate internal structures and are made from superior material constituents. However, there are issues of contaminants (intracrystalline proteins that accompany biogenic crystals) and lengthy biodegradation properties. In addition, there are questions over the ecological impact of stealing marine biostructures away from their natural habitats. It is possible to temper the impact and implications of doing this by implementing the latest updates in farming techniques and growing these marine structures in artificial aquaria [69]. There have been excellent studies aimed towards effectively growing corals and marine sponges in natural habitats in fitting quantities and with the qualities fit for commercial activities and biomedical purposes. In one case, marine sponges have been farmed with the express purpose of producing bioactive metabolites in practical amounts [70] Cloning of the gene coding for the metabolite of interest has been advanced as a solution for this that can be sustained for all needs and applications [71,72]. Similarly, there are options for coral skeletal supplies in guaranteed quantities [73]. Having rapid growth rates of 2.2 mm/month at 18 °C, calcareous algae, *Corallina officinalis*, may provide more sustained volumes of structures compared with corals, which exhibit an accretion rate of 10 g/m^2^, to the future hard tissue engineer [74,75]. Cuttlefish bone, sea urchin spines, and seashells have all been tested and evaluated to varying degrees as potential structures to support bone formation [68].

**Figure 2:**
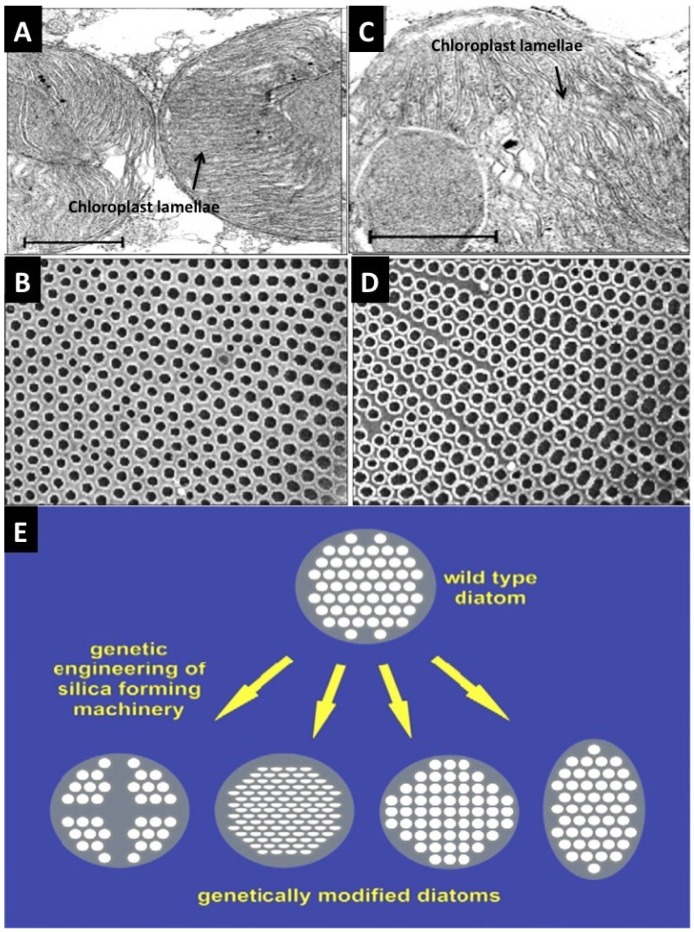
Ideas for re-design of the structure of microshells via genetic and metabolic routes. The architectural features of *Coscinodiscus wailesii* species of Diatom frustules can be changed via modulation of the chemical and biochemical composition of the growing environment [77]. (**A**) TEM cross-section through chloroplasts in Diatom cell to show the normal ultrastructure of stacked lamellae. This exact arrangement of organelles molds the areas where silica is deposited and hardened to produce a regular externalized pore structure (**B**); (**C**,**D**) Added Nickel sulfate-in “sub-lethal quantities”-in seawater distorts the shape and packing arrangement of lamellae, which in turn affects silica pattern formation at the cell surface. The result of this is to double the pore size and increases the packing density of the pores. This property is something we want to exploit. Gene modulation of *Thalassiosira pseudonana* has led to the directed evolution of differently structured Diatoms with stark variations in pore size and patterns of pores [78]. This can be a basis for adapting inorganic structures and architectures to desired formats.

One other way to obtain organism-built structures and one that minimizes habitat degradation and supply issues is to cultivate the organism of interest in clean and strictly controlled conditions in the laboratory to grow structures for a particular purpose. This promotes the idea of manipulating an organism—Its physiology, genetic coding and perhaps epigenetics—To generate mineralized structures according to the experimenters’ specifications of architecture, shape, size and morphologies by direct modulation in real-time. There are also possibilities to supplement the developing and growing structures with choice additives. These additives can be included in the growth media. For example, the living diatom cell uses silica transporters to shift any excess amount of molecules found in its environment directly into the developing frustule. This system has begun to be manipulated in the lab. In the case of a genetic engineering approach to “designoid” inorganic structures, there have been few concrete examples [76]. Metabolic modulation of growing Diatoms can exert alterations in shell architecture [77]. Gene modifications enacted on the diatom *Thalassiosira pseudonana* genome have led to adaptations in shell structure in just a few generations [78]. Thus far, the gene modulation process can be used to create gene knockouts and RNA-interfering inserts into the genetic constitution of diatoms (Figure 2).

### 2.2. Marine Products in Dental Tissue Engineering

**Figure 3:**
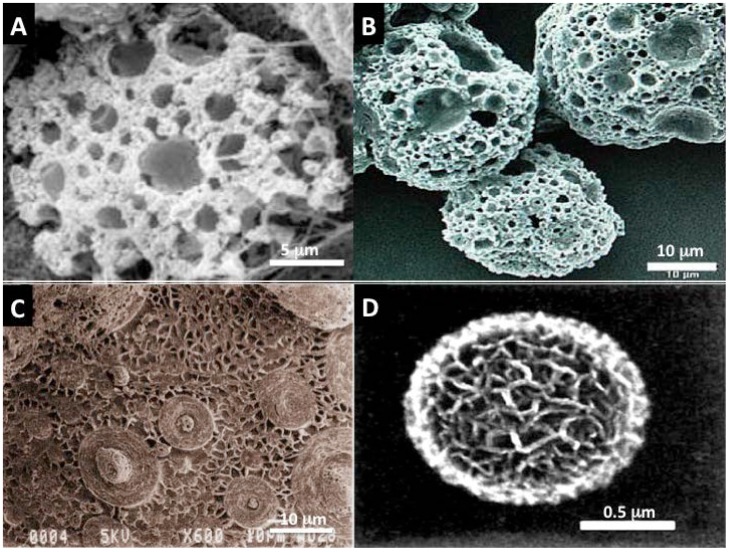
Simple inorganic “designoid” objects [76] in nature recreated in the beaker using biomimetic template mediated materials chemistry (**A**,**B**) Mesoporous vaterite microsponges produced via mineralization at the interface of a SDS stabilized water microdroplet in oil; Such structures have also been manufactured using plant based surfactants and oils. The stable emulsion is reminiscent of a “viniagrette“ [83,84]; (**C**) Inorganic surface with multiple patterns that are strongly reminiscent of Diatoms and *Radiolaria* shells, created by heat treated mesolamellar aluminophosphate vesicles [80]; (**D**) A single mesoporous sphere of aragonite polymorph of calcium carbonate. The principle behind its formation is analogous to the arrangement of areolar vesicles in diatom mineralization. A thin soapy foam is created from an oil and water mixture. This serves as a template for calcium carbonate mineralization [80]. Using some basic construction principles learnt from nature such as: make vesicles and pack them together like foams and use them as boundaries for mineralization to build on, it is then possible to generate some of the structures and architectural motifs seen amongst *Coccolithophores* and *Foraminifera* shells [80,81,83]. These microsponges by virtue of their shape and structure have been successful in drug and gene delivery experiments and in 3-dimensional co-culture with human bone marrow stromal cells.

The marine environment is filled with biodiversity that extends into many levels, chemical and structural. In the past, marine organisms have been examined for their potential to yield new drugs, adhesives and filters. Biomaterials and skeletal structures are relatively recent products that biotechnologists and, to a limited extent, their tissue engineering counterparts have used [36,37,73,79]. Some have been made with strong biomimetic principles (Figure 3A–D). The marine world of structures has also been used as a template for recreating copies of some basic skeletons (inspired mechanistically by the intricate skeletons of *Foraminifera*, *Radiolaria*, and *Coccolithophores*) and for creating new emerging morphological forms [80,81,82,83] (Figure 3A–D).

There are two valid ways of potentially implementing these structures into the tooth. The first method is to deploy them without cellular contents: to implant a structure infused with regenerative agents into the site of tissue loss and allow the interplay with host biology [84,85]. This approach is designed to lead to the growth and colonization of endogenous tissue throughout the entirety of the construct on the understanding that the local repair and regenerative responses are readied and powerful enough to carry this through. A second option is to use the structures to grow mature tissues in culture that are ready to join and integrate with identical host tissues following transplantation and engraftment [25,86].

The possible products that can be employed in dental tissue engineering include marine sponge skeletons, nacre seashell, Diatom frustules, *Foraminifera* shells, and coral skeletons [36,38,39,54,87]. A vast number of materials and substrates are being investigated, including marine collagens, alginates from seaweed, chitosans, chitins, and chito-oligosaccharides from crustacean shells. These substrates have been published extensively elsewhere [88,89,90,91,92,93,94,95,96,97,98,99,100]. However, there are some less well known and more recent marine-based materials and substrates that have been experimentally implemented in dental hard tissue restoration, cleaning and augmentation roles. This illustrates the wide variety of substances available from life in the oceans.

#### 2.2.1. Tooth Remineralization

Methods for *in situ* tooth remineralization are becoming more diverse and follow biomimetic and bioinspired directions. Inorganic nanoparticles are viewed as fundamental building blocks and seedlings for the emergence of new dentine and enamel crystals with correct shapes, sizes and axes [62,63,101,102,103]. Overall, these items are an attractive proposition because of their low invasiveness and conceptual simplicity; there is no need for surgery or the inclusion of traditional restoration materials. However, there has been little actual success in formulating substrates that remineralized in place inside the mouth, which is not a constant mineral-enriched environment as all of the *in vitro* tests imply. One mechanism that possesses the right virtues for stabilization at the tooth surface in the mouth is polydopamine-induced hydroxyapatite formation, which is reported to grow crystals in the image of its surrounding mineralized environment. The main component of mussel glue (e.g., *Mytilus edulis* foot protein-5), which is calcifies onto marine substrates, could adhere to the tooth surface and successfully remineralized etched-in defects [104].

In addition to mussel glue, nacre organic matrix extract has also been used for enamel remineralization. The sophisticated hierarchical structure of human enamel and the construction rules for building it are all well understood, but as yet, materials scientists are unable to match this structure. Artificially created nanocrystals that mimic the size and shape of enamel nanocrystals have been shown to induce enamel reproduction at the tooth surface in the beaker. However, these nanocrystals are not clinically viable because they lack stability inside a defect, are less tough than real enamel and mineralize too slowly and would be washed away in the mouth [62,63,105]. Hydrophobic protein amelogenin is the principal matrix protein that helps shape the crystals of enamel in the early developmental steps [106,107,108]. Mature enamel is structured by acidic enamelin glycoproteins that coat each individual crystal [83,109]. In one surprising lateral study, a very different type of mineral-building protein from the organic matrix protein of nacre fraction could also shape and pack hydroxyapatite crystals in the exact same parallel bundled orientation as seen in normal enamel and that this translated into a degree of hardness comparable with natural enamel [110]. Perhaps this correspondence in actual enamel and analogous enamel mineralization represents the evolutionary conservation of molecules (proteins) involved continuously throughout the history of biomineralization. Since the water soluble matrix of *Pinctada maxima* nacre is purported to contain TGF-β class of signaling proteins that can participate in bone remineralization [111,112]. We have seen further accumulating examples of this across evolutionary time and space [69,113,114,115].

#### 2.2.2. Biofilm Prevention and Disruption

Marine organisms are in a constant *arms race* to counteract attacks by predators and parasites. Many organisms have sophisticated, mainly chemical, defenses to fend of these attackers. Bacterial biofilms pose a threat, and many organisms have developed defenses to disrupt these biofilms from taking hold [115]. This battle also exists between different bacteria and other micro-organisms. They themselves have been equipped with chemical bioagents that prevent colonization of competitors [116]. Under intense scrutiny right now are polysaccharides from marine bacteria and other organisms such as, marine sponges that perform disruption of biofilms [117,118]. In a special finding during an investigation into shifting films on seaweeds, the bacterial biofilms formed by a ubiquitous *Bacillus licheniformis* released an enzyme that allowed bacterial dispersal from the embedding medium of the biofilm. The NucB enzyme involved in this process is being developed to disrupt biofilms on the tooth surface after the successful disruption of the biofilms that cause sinusitis [119,120]. However, it remains to be seen whether this enzyme is generic and can effectively dislocate the biofilm structures secreted by other species. The surfaces of marine organisms larger than a single cell are under a constant threat of attack and colonial settlement from microorganisms and parasites. In addition to chemical defenses, organisms possess structural defenses that have evolved to combat bacterial contamination of the outer skin surfaces that can cause physical damage to tissues. One of the best-studied structure-based adaptations is sharkskin, although there are undoubtedly more examples of this phenomenon in other marine organisms [121,122]. Nature has evolved countless interfaces with anti-bacterial defenses using specific nanotopographies. This surface defense is independent of the effects of chemical secretions. The first application of patterned surfaces aimed at biofilm control resembles diamond-shaped protuberances at the micrometric scale that hinder bacterial contamination. The engineered version is called Skarklet™ and is inspired directly from the micron structure of the scales or dermal denticles of sharkskin [122]. The topography is thought to increase the surface energy at the surface to such a degree that it prevents the expenditure of excessive amounts of energy by bacterial colonies needed to generate a biofilm at the surface. Bacteria die unable to connect with other bacteria cells [122]. The effect of this material has been evaluated in a medicine-relevant study [123]. The synthetically replicated surface hindered the growth of *Staphylococcus aureus*, *Staphylococcus epidermidis*, Methicillin-resistant *Staphylococcus aureus* (MRSA), *Pseudomonas aeruginosa*, *Escherichia coli* and vancomycin-resistant enterococcus (VRE).

#### 2.2.3. Scaffolds and Biostructures

##### 2.2.3.1. Marine Sponge Skeletons

**Figure 4:**
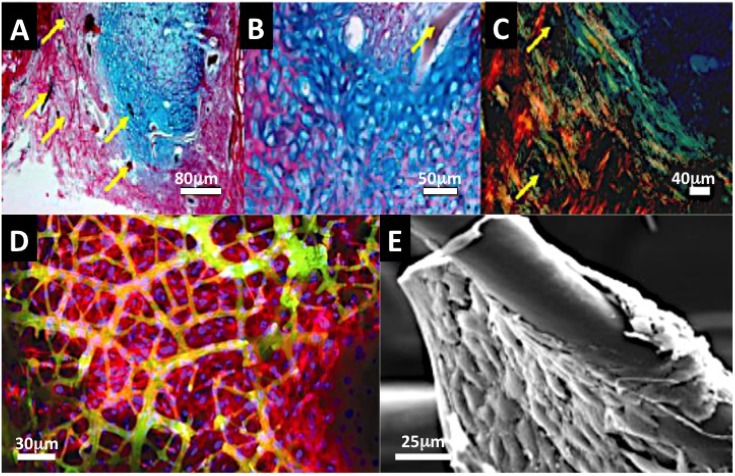
Collagenous Marine sponge comprises of a fibrous framework of bonded fibers and this could be an ideal substitute for a periodontal ligament and bone tissue (**A**–**C**). The advantage of selected marine sponge skeletons over many synthetic fiber networks and electrospun constructs is that they are fully fused together. In (**A**) we see rapid colonisation, proliferation of periodontal ligament stem cells on a collagenous marine sponge within 48 h of dynamic seeding (×200). The PDL stem cells were centrifuged into the marine sponge framework at 1200 rpm for minutes. The fibers provide surfaces for periodontal ligament stem cells to develop a stretched morphology, which is characteristic of their home environment and they span large spaces existing between sponge fibers in all directions. An early form of bone and cartilage tissue is developed *in vivo* within a collagenous marine sponge skeleton from seeded embryonic stem cells (**B**,**C**). The histoarchitecture is early in origin and the organisation is characteristic of osteoid type of bone and cartilage. The bone that was formed showed highly organized matrix when viewed under polarized light, hinting at the possibility of highly organized mineralization in progress (Yellow arrows point to sponge fiber) (4**A**–**C** Kindly reprinted from Biomedical Materials [38]); (**D**) Confocal fluorescence image of osteoblast cell sheets attached and suspended in marine sponge framework at 14 days. The top panel showing cell distribution at high power and the lower panel showing coverage across the whole seeded marine sponge skeleton (red = F-actin staining; green = autofluorescence of sponge fiber; blue = nucleus staining); (**E**) SEM image of osteoblast cell aggregation on *Hippospongia* fiber; (Figure D was reproduced with kind permission from Ivyspring International Publisher [54] and Figure E reproduced with kind permission from Wiley-VCH [129]).

The collagenous skeletons of marine sponges have been shown to be effective frameworks supporting a range of cells and tissues and in particular bone tissue [36,54]. The skeletons provide 3-dimensional spaces for cell or tissue colonization and a level of tissue pre-organization. There are sufficient vital structural cues and some other biochemical cues that are able to support and maintain bone and cartilage tissues. The void volume is large, approaching 1000 μm, and is interconnected in all directions. However, the pore gaps are greater than the optimal sizes usually put forward for a prospective tissue colonizing sponge framework. In truth a range of pore sizes are thought necessary to transport fluids, proteins and for cell migration to occur. Typically the optimal pore sizes for cell migration are between 80 and 500 μm [124]. However, the size range is equal to the size of structure for an essential functional unit of tissue [125]. The sponge fibers are fully bonded, and the mixture of collagens blended into the fibers is constitutively made up from analogues (short chain) of basement membrane collagens, such as collagen type IV [126,127]. In one specified marine sponge a type 1 mammalian analogue has been discovered [128]. Initial observations suggested that MSCs hone to fibers, attach and spread even when cultured in serum-free media [36]. We observe the exactly the same response with human Periodontal ligament stem cells (low passage number) cultivated within a *Spongia* collagenous Marine Sponge (unpublished data). Within an animal model, the subcutaneous responses are dramatic when fetal derived mesenchymal stem cells are embedded in a *Spongia* collagenous sea sponge for 3 weeks (Figure 4A–C). In a separate *in vitro* study osteoblasts cultured within *Calliospongia* framework progressively anchored onto the fibers, expressed alkaline phosphatase and within 3 weeks generated mineral nodules and expressed mid to late osteogenic genes, osteonectin and osteopontin [54] (Figure 4D). In a paper by Zheng *et al.* marine sponges from the genus, *Hippospongia* and the family *Chalinidae* supported mouse osteoblast adhesion, growth and proliferation [129] (Figure 4E). There is also active development of tissue engineering matrices using silica, chitin and collagens from marine sponges [130,131]. The potential of this framework through careful and systematic studies seems promising in bone and the periodontal ligament junction with bone and dentine.

##### 2.2.3.2. Diatom Skeletons

**Figure 5:**
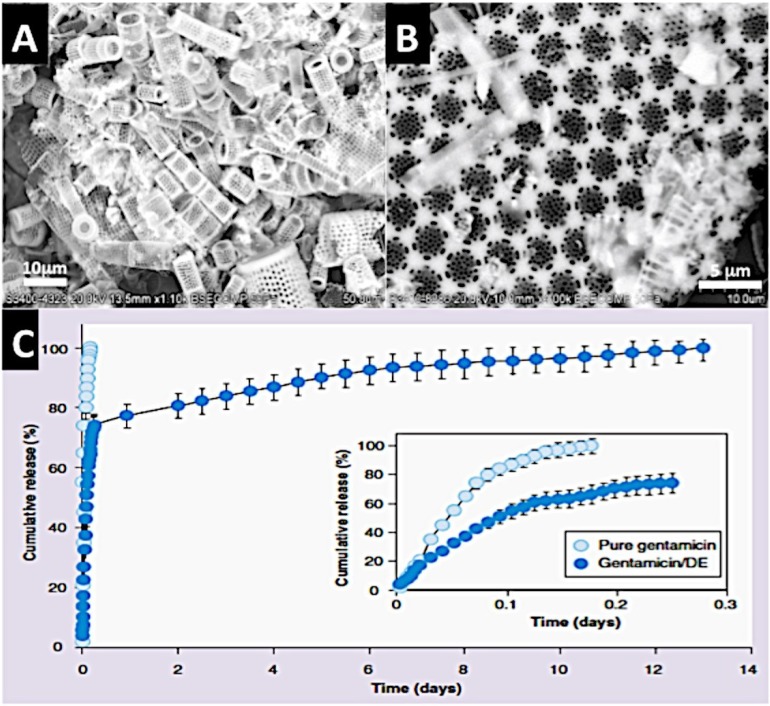
A Diatom shell structure that can deliver incorporated antibiotic. (**A**) A low power SEM of one species of Diatom shell (cylindrical *Aulacoseira* sp.) from diatomaceous earth; (**B**) High power SEM of a Diatom shell to show the nanopores incorporated into the microstructure of discoid *Coscinodiscus wailesii*; (**C**) A cumulative release profile for shell incorporated Gentamicin antibiotic lasting up to 14 days before all of the product has been off-loaded. This demonstrates that Diatom shells possess a potential for controlled drug delivery (C kindly reprinted with permission from Future Medicine) [132].

These skeletons are showing promise as efficient drug and protein delivery devices [132], an additive for dental Zirconia nanocomposites [133], as a tissue engineering “space filler” and framework and a model for cell encapsulation [134]. Diatoms are microscopic algal cells generate highly intricate shells made of silica glass. The structures are both a small filtration device and an armor plating to protect the organism from predators. These structures possess features and traits that can be exploited for a similar function but for a different set of purposes. Thus, they can be matched to a very different role than that for which they evolved. Diatomaceous earth is a widely used mineral constituent for many industrial applications. In Dentistry it has long been a routine additive for impression materials. Diatom skeletons have been used in technologies spanning sensor devices to paints and now extended vigorously into nanotechnology and biotechnology fields [135,136,137,138,139,140,141,142]. The structures possess an adaptive fitness within hard tissues, dentine, bone and enamel because of their toughness, hardness and mineral compatibility with calcium phosphates [143,144,145,146]. Such shells are being adapted for drug delivery [132,147,148] and biosensing roles [149]. We have proposed to use them as agents for cell recruitment, proliferation and migration and as a packing powder for dentine defect filling, and a completed regeneration of mature mineralized tissue. A prime objective would be to reproduce dentine structure in its primary state with high bioceramic fraction and with tubules. Faithful replication of dentine properties can be achieved through chemical self-organization and assembly or via the activities of incorporated dental cells such as, dental pulp stem cells. The primary function of Diatom shells in a tissue engineering context is as a protective container for proteins in cell activation during transplantation and regeneration. The pore sizes range from microscopic to nanoscopic dimensions (200 nm to 1000 nm), which provide the fine capillaries for drawing in soluble proteins and holding them within a tight network (Figure 5A,B). There are two ways that proteins are stably entrapped in the Diatom skeleton. One is via chemical adsorption and the other is by adsorption via weak, low energy van der Waals forces [132]. The electric charge of the protein and its molecular size determine the encapsulation properties, stability and rates of release from the structure. Diatom shells elute proteins at a slower pace compared with comparable hollow and porous particles made from silica. The release display is time dependent and occurs at a constant rate, thereby representing zero-order kinetics as shown for incorporated Gentamicin sulfate in Figure 5C. Mesoporous silica particles are favored to pack in many different agents for a variety of biological roles such as tumor destruction via the release of anticancer drugs and gene delivery inside the cell [150,151]. Today, these processes can be more tightly controlled with surface molecule decoration [152]. The smallest varieties (80–200 nm) can enter the cell without endangering it. The sizes of the pores and the surface chemistry of the pores can be delimited with accuracy. Being able to specify channel orientation and the size of individual domains will help the control in the precision of encapsulated delivery. Thus, it becomes more realistic to tune the rate, regionality and timing of any particular encapsulated protein.

The dentist requires a flowable paste that effectively fills all the spaces, including cracks and fissures within any deep dentine defect. So, we have begun to fabricate varying formulations of Diatom containing pastes that rapidly harden in place in the entire space. Some of the pastes have added to them: calcium phosphate mineral precursors, dental pulp stem cells and phospholipids. In our laboratory we have made mineralized structures with pores similar to normal dentine and we have shown high survival and proliferation rates of Dental pulp stem cells embedded with Diatom shells and an alginate gel. Mechanically, the hardened and crystallized paste containing 75% w/v diatoms behaves as a shock absorber when compressed (at extremely mild loads compared to normal dentine) and yield dramatically more than native dentine. The idea is to provide a shape and void filling mass filled with dental stem cells that supports and cushions an added metal filling above-representing the enamel substitute. Our aim is to keep this as a short-lived transitional structure until cell-originated dentine tissue is reproduced and becomes more densified as calcium phosphate mineral is secreted from a developing dental cell population, such as DPSC, deposited and is remodeled into a proper, anatomically correct dentine structure.

There are several ways to grow microscopic diatom shells on culture dishes in the laboratory for the re-design and partial re-engineering of their structure and architecture. Having control of the conditions in which the shells grow and the composition of their growing media, it becomes possible to alter the pore diameter and architecture such as, the geometrical arrangement of the pores. It has been demonstrated that any new additive in the culture media is gathered into the molecular machinery of skeleton formation. The result is that the inclusion is infused into the amorphous silica framework. Doping of the skeleton is possible because the processing pathways in silica deposition materials chemistry are predisposed to picking up molecules from the local external environment.

##### 2.2.3.3. *Foraminifera* Microskeletons

**Figure 6:**
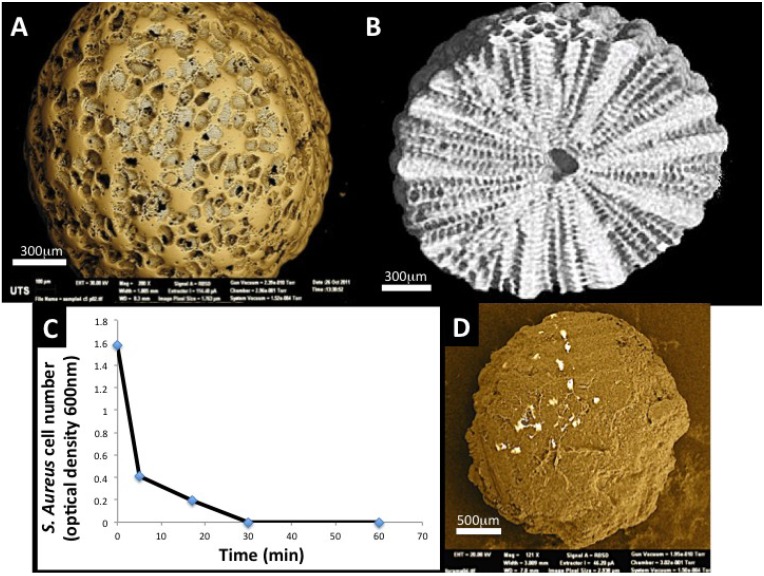
*Foraminifera* spheres are macroscopic shells from marine sediments that grow a highly intricate set of pore structures for the task of passive filtration where food particles are sucked in through channels and by design selected into sizes for the cell inside; (**A**) The natural structure of interconnected pores and channels has shown a usefulness in tissue engineering for capturing and slowly releasing bone building drugs and supporting proliferation and expansion of stem cells around the outer surface in 3-dimensions (Image generated at the Microstructural Analysis Unit UTS, Sydney, Australia); (**B**) Close-in cross-sectional view of the macrosphere to show the regular and highly ordered distribution of pores inside the sphere. This image was generated by micro-Tomography (micro-CT at the Australian Centre for Microscopy and Microanalysis); (**C**) A look at the functional release of Gentamicin antibiotic from macrospheres on the eradication of *S. aureus* bacteria. At 30 min all bacteria cultured with Gentamicin laden *Foraminifera* macrospheres were exterminated; (**D**) An SEM image of a single macrosphere coated with rat adipocyte derived stem cells (ADSC) 7 days after seeding (C Reproduced with kind permission from Future Medicine and amended to improve clarity in reproduction [156] and D Reproduced with kind permission from Anne-Liebert [79]).

*Foraminifera* shells are strong contenders as bioactive bone substitutes, drug delivery devices and as an equivalent of bone allograft. Naturally occurring microskeletons are produced by hundreds of thousands of unicellular amoeba like Protists that predominantly reside in all marine habitats throughout the globe. They are in such abundance that one third of the earth surface is coated with dead shells from these primitive organisms. Large benthic, coral reef dwelling *Foraminifera* shells, without their host creators, can be found deposited in coral beach sands on reef flats and sand cays representing 40% of the total beach sediment. Large benthic *Foraminifera* have been collected from Bali, Indonesia and exploited in studies for bone healing therapies including osteoporosis [153]. Structurally, these microskeletons are made up of an intricate networking of micro- and nanopores, evolved by natural selection as a particle filtration system as seen under SEM and μCT (Figure 6A,B). These structural properties have been effectively exploited for sustained, targeted drug (bisphosphonate) and antibiotics delivery (Gentamicin) with low toxicity [154]. An additional level of control in delivery is implemented by wrapping up individual spheres with liposome bound coating [154]. The shells are well suited as filler particles in calcified tissue environment because of their mineral content and structure. Hardening the shells by thermal transformation from their original calcium carbonate identity to a calcium phosphate one coupled with an additional nanometric overcoat ensures that the shells can be compacted into bone voids without the loss of the important structure. Antibiotic compounds have been encapsulated into the pores and onto the surface as crystals with 73% loading efficiency. The drug is released gradually with the concomitant dissolution of the mineral. In tests with *Staphylococcus aureus*, co-cultured macrospheres infused with gentamicin were completely eliminated in 30 min (Figure 6C). Other important bone induction drugs have been offloaded from these efficient structures to increase bone formation and they can reduce the toxic side effects of drugs because of the controlled targeting. Simvastatin-loaded macrospheres exhibited superior effects on bone growth and strength *in vivo* during prolonged release over 6 weeks compared with control samples in OVX mice [155]. Repeatedly, we see the possibilities of controlled release properties for bone induction agents and the added effect of the release of biogenic ions from the structure upon dissolution. The inductive effects on bone cells of strontium, calcium and magnesium ions released, at physiologically relevant quantities have also been studied [39]. The ions have been highly effective at promoting activities of a bone-secreting cell while at the same time suppressing the activity of bone resorbing osteoclast-like cells in the culture dish [79,154]. Thus, the macrospheres possess an intrinsic property for bone homeostasis.

The idea of using carrier particles to support proliferation of cells has been around for some time. *Foraminifera* macrospheres have potential to support and proliferate cells in 3-day very rapidly. In one simple test of this, the seeding of Adipocyte derived stem cells (ADSC’s) into calcium phosphate transformed *Foraminifera* shells via centrifugation leads to the complete coverage of individual spheres in 7 days via a steady increase in proliferation rate and an increase in secreted matrix proteins [79] (Figure 6D). This result demonstrates how the spheres can be effective as templates for cell accretion in 3-dimensional culture regimes. The expansion of stem cells in the culture dish is one of the important steps towards increasing availability for cell therapies. Thus far, we have merely harnessed these structures in the first steps towards re-building mature bone inside the voids created by disease and traumatic injuries.

#### 2.2.4. Bone Substitutes

##### 2.2.4.1. Nacre Seashell

Nacre seashell has shown intermittent promise as an osteoinductive bone substitute. The complete story of nacre osteoinduction and its potency has not been revealed and there are still opportunities to further exploit this material for use in oral surgery for example particularly when coupled with bone marrow derived mesenchymal stem cells.

**Figure 7:**
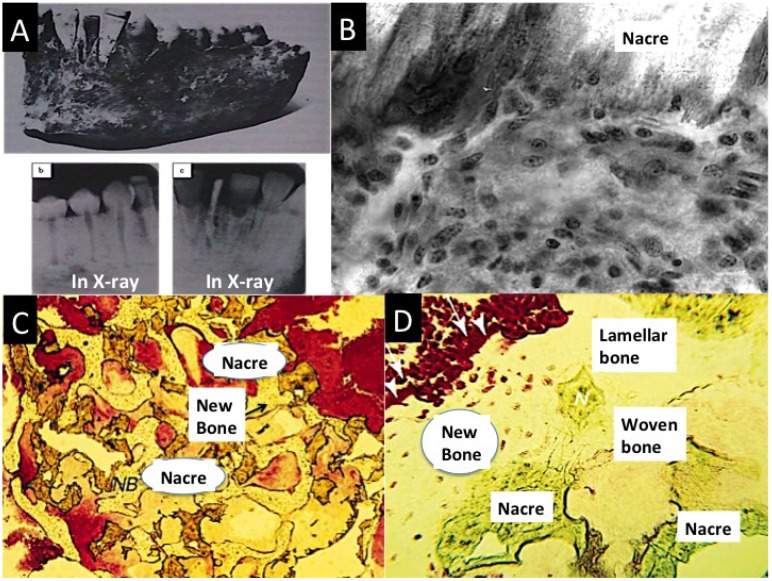
(**A**) Whole nacre incisor replacements found in the lower jaw of an Ancient Mayan individual. Excellent bone fusion is shown by X-ray imaging in some of the nacre implants in panels, b and c; (**B**) A high power (×600) image of a section at the interface between a nacre piece and metabolically active mesenchymal cells mobilizing into an osteoprogenitor layer. The junction is free of immune cells are present and fibrous tissue [162]; (**C**) Stained histological sections from human patient biopsy showing the integration between bone and nacre coupled with new bone formation; (**D**) A high power view of new bone at the nacre surface showing particularly the stained osteoid borders. Also generated are two textures of mature human bone: woven and lamellar; (Plate A, reprinted with kind permission from Bulletin of the History of Dentistry, Copyright (1972) [160]; Plate B reproduced with kind permission of Elsevier [162]. Plates C,D Reproduced with kind permission from Macmillan Publishing Group [115]).

The pearly outer layer of seashells in mollusks is nacre. This structural biomaterial has long been subject to study for its superior mechanical strength even when wet (Young’s modulus 60 GPa), which is equivalent to the strength of steel [157]. The fascination for this material is how a brittle ceramic gathered from the marine environment can be made so tough into seashell with a tiny, 0.24% fraction of bioorganic material. Nacre is riddled with fracture-deflecting features that include: advanced ductility by inclusion of water and superior molecular interlinks between the platelets and the matrix into which they are embedded. Nacre is a prime example in which cheap soft materials are exactly structured into a new material that has superior mechanical design and strength. Materials scientists have spent many years striving to emulate the way nacre is built to make stronger and tougher materials using cheap, abundant, soft, unelaborated, mechanically weak starting materials. According to the community of materials technologists, the greatest present and future need is for energy resourcefulness [158]. Nacre, quite by happenstance, may have a significant role to play in mammalian biological tissues because it was discovered that nacre has a bone-forming property [111,112,159]. There is a deep historical perspective to this story as in 1931 Mayan skulls were found to have pure nacre teeth which had fused perfectly with the jawbone [115,160] (Figure 7A). On closer investigation, there is a rational reason for this connection. Molecular processes in biomineralizsation have been conserved throughout evolution, and some key components are found in both invertebrates and human mineralized tissues and have the same or other adaptive functions.

The vital constituents of nacre in biological mineralization in bone tissue are the water-soluble matrix proteins. Powders and small chips made from nacre seashell have been used to fill bone voids. This was undertaken based on the knowledge gained from one of the first studies to study nacre led bone mineralization. Nacre chips were found to co-exist with bone chips and eventual weld together with newly formed woven type bone in the presence of human mesenchymal cells, osteoprogenitors and osteoblasts *in vitro* [161,162] (Figure 7B). Vitalised by this finding, in one of the first experiments undertaken with this biomaterial in a human patient, nacre powder mixed into a slurry using patient’s blood was injected into a jawbone defect [159] (Figure 7C). There was no inflammation and fresh bone had been generated as the nacre resorbed. Other subsequent experiments have tested nacre’s osteogenic power in few laboratory animals, such as sheep and have reported similar dramatic osteostimulatory effects [163]. In 12 weeks a defect excavated in the lumbar vertebra and filled with nacre powder transformed into spongy bone while the nacre was seen to have dissolved [163]. In comprehending these results we cannot rule out some osteogenic effect from the nacre surface structure itself such as the micro- and nanotopography. In addition, there is proper evidence which shows that the surface chemistry changes at the surface of nacre [164].

Nacre organic matrix proteins, but principally peptides have been demonstrated as the origin of osteoinduction. In the study by Lamghari *et al.* it was proposed that the matrix peptides stimulated cellular responses that would be expected from treatment with Bone Morphogenic Proteins (BMP’s) and Transforming Growth Factor- beta (TGF-β) [163]. The biochemical responses of bone marrow stromal cells (hBMSC) to nacre for instance are *like-for-like* following treatment with nanogram quantities of TGF-β. TGF-β is a central protein in dentine building in embryonic development, and it is speculated that TGF-β could play a role in dentine regeneration in adults. The role of BMP in the tooth has been intensively studied.

**Figure 8:**
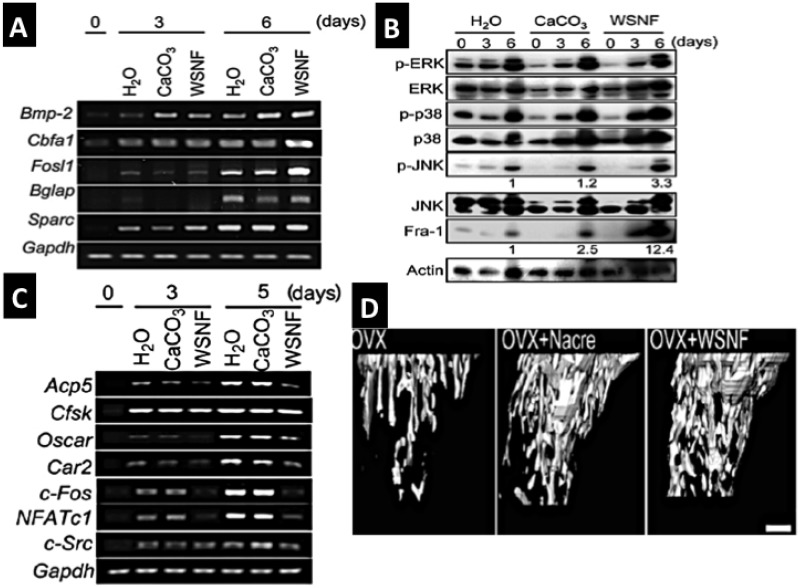
The effectiveness of nacre WSM in bone formation, remodeling potential and osteoporosis treatment; (**A**,**B**) Nacre powder and WSM extract induce osteoblast mineralization via consequential molecular signaling pathways e.g., Fos-1, p-ERK, p-JUNK and up-regulate biomineralisation promoting genes BMP-2, cbfa-1 *etc.* Osteoclast gene activities are suppressed (**C**). Nacre products also prevent osteoporotic bone in OVX mice (**D**) (A–D reproduced with kind permission from Elsevier) [165].

A series of efforts have been made to extract, purify and concentrate the key regenerative proteins into a soluble preparation having precisely determined biological effects on different cells acting in bone biomineralizsation. A non-mineralizing route towards extraction of the intrinsic matrix proteins has been asserted as the best way of isolating the osteoinductive constituents rather than a mineralizing mode of extraction [111]. The extracted and concentrated preparation of water-soluble matrix (WSM) has shown considerable potency. The protein extract boosts proliferation and ALP secretion in bone marrow cells in high microgram quantities (135 and 540 μg protein/mL) [112]. It has been shown to induce osteoblast driven mineralization activities much faster than when osteoblasts are placed in a mineralizing medium [166]. Very significant results have come from the research of Kim *et al.* [165] In it nacre WSM extract was demonstrated to effect molecular signaling pathways and regulate gene expression activities differently in osteoblasts and osteoclasts. Osteoblasts were promoted and osteoclasts suppressed in the presence of WSM strongly implicating the importance of nacre in bone remodeling process (Figure 8A–D).

Thus far, 19 protein fractions have been disclosed via size-exclusion HPLC relating to 110 individual molecules being present. A 60% proportion of the WSM protein is under 1 kDa meaning that they are probably mainly peptides [167]. An earlier study highlighted 4 main fractions with one in particular having the same power of osteoinduction as BMP-2.

There is no currently available data featuring nacre-driven mineralization via a cell participation-route in hard dental tissues such as, dentine or enamel, but there is an intriguing study in which tooth slices having the enamel etched away would re-mineralize into almost perfect enamel structure when placed in a solution containing extracted water soluble matrix of nacre (WSM). The newly re-mineralized enamel analogue consisted of hydroxyapatite rods tightly packed together into well-ordered bundles. [110]. Nacre WSM appeared to provide a template for Hydroxyapatite rods and also glued them together into the tight bundles.

##### 2.2.4.2. Biotransformed Coral Skeletons

**Figure 9:**
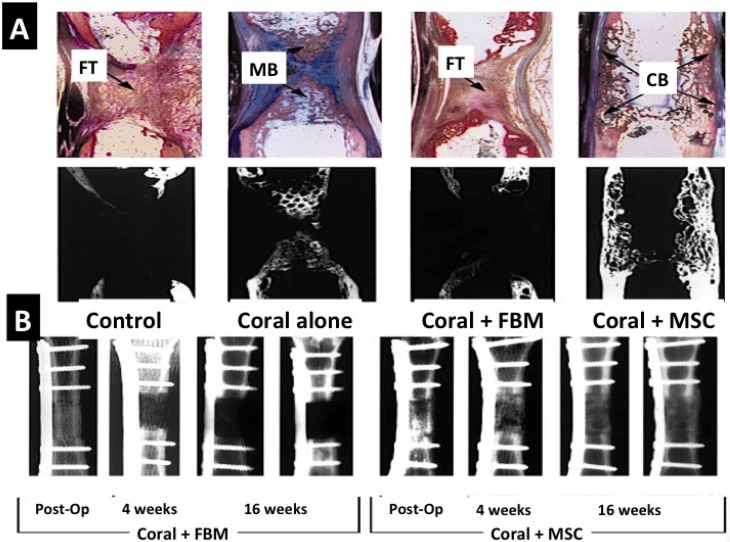
Live animal bone repair using coral skeletons (untransformed) laden with mesenchymal stem cells. (**A**) A comparison of host bone reactions in histological stained section to a segmental defect (control), a segmental defect plugged with coral skeleton (Coral alone), plugged with coral skeleton and FBM and Coral skeleton filled with MSCs. In the group containing coral with MSCs the defect is bridged and repaired in full thickness; (**B**) X-ray slides comparing the mineralized bone replacement between coral skeleton with FBM and coral skeleton laden with MSCs. Only in the corals with MSCs do we get new mature bone replacement (Reprinted with kind permission from MacMillan Publishing Group) [175].

Coral skeletons have been used as bone substitutes in surgery for the last 28 years but it was not until the 1990s that it became commercially available as Biocoral and Interpore [168,169,170,171,172]. A pioneering idea for harnessing coralline materials in medicine came from White *et al.* in 1972 with an innovative process for transforming and generating high fidelity copies of marine skeletons such as coral [51]. Further studies were carried out to assess tissue growth responses to these new implants [49,50,51]. The first study in which a treated and transformed coral was used in a clinical application happened in 1985 [173]. Now there is a substantial catalogue of published studies attesting to various levels of clinical efficacy to heal widely separated fractures and fill naturally unbridgeable bone voids caused by tumors for example [174]. Bone tissue morphogenesis has been augmented within coral skeletal frameworks by incorporating bone marrow derived stem cells with impressive outcomes (Figure 9A,B). In a large animal defect model mature cortical bone was produced inside coral frameworks housing MSC [175]. Owing to their internal pore and channel structures, crystallinity and composition corals are biomimetic substitutes for different textures of bone but only two principal classes of reef-building coral skeleton are actually used (*Porites*, *Acropora* and *Goniopora*) because they are available in large quantities and possess a highly consistent structure [176]. Their immunogenicity is negligible when thermally treated with only tiny quantities of intracrystalline proteins remaining. Coral skeleton implants are currently limited to non-load bearing regions of the skeletal system as they possess inherent weakness in compression. This use has spurred methods to increase the strength of coral skeletons so that they can support the high compressive forces exerted in load bearing long bones for example. One major way of increasing strength in compression of coral skeleton bone grafts is to chemically transform them from their native calcium carbonate composition into hydroxyapatite. Commercially produced converted coral skeletons for surgery are not completely converted into hydroxyapatite so that some parts will remain as calcium carbonate. Further strengthening is achieved by delivering hydroxyapatite based sol-gel nanocoatings onto the entire surface of struts and connectors and into the coral micropores and nanopores [177]. The combined appropriation of the calcified material by heat, transformation into calcium phosphate mineral, nanocoating and pore infilling doubles the biaxial strength compared to untreated normal coral [177]. It has been shown that an equivalent alkoxide-based hydroxyapatite layer on titanium discs is a direct stimulant for osteoaccelerants, c-fos and Map kinase pathways in attached osteoblasts [178].

In our own laboratory we have exploited and appropriated isolated and selected structures from marine bioresources on the basis of biomimetic logic. There are other ways of doing biomimicry but we have described a facile approach with good clinical potentials. Corals exhibit a structure and composition equivalent to human bone, and the spherical filtration devices have the properties necessary for drug encapsulation and delivery. These biostructures have been selected purposefully and simply by observing the structure and knowing something of its function. In partial reconstruction of tooth tissues, it is frequently necessary to build more than one single tissue because there is a gradation of tissue types. For instance, a periodontal ligament substitute will need more than a fibrous web as well as intermediate interfaces for connection with bone and dentine, respectively. Therefore, because the structures are only temporary, we must think of reconfiguring and appropriating such structures to emulate this feature or to build zones of distinct tissues or cellular zones that are fashioned with specific mixes of cell phenotypes and extracellular matrix (ECM) compositions.

**Figure 10:**
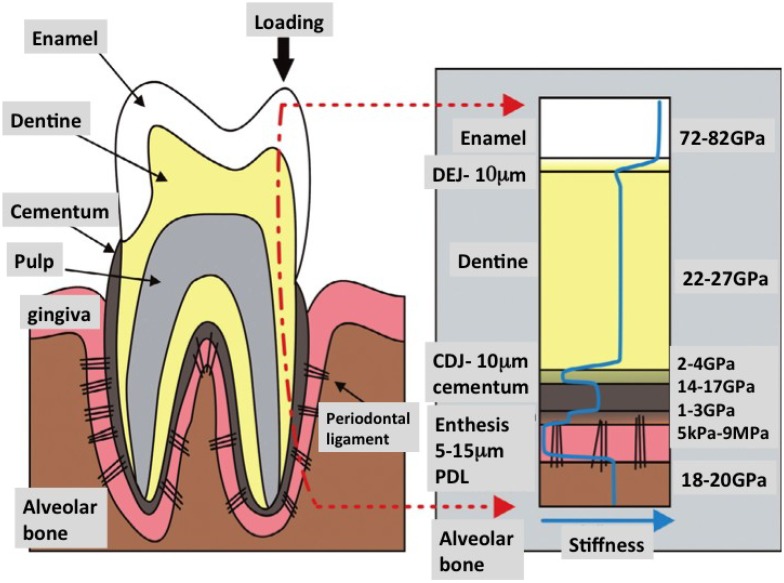
In building living tooth replacement tissues, addressing issues of mechanical design is paramount. The most potent lesson to learn from nature is the design of smart interfaces. In the right panel is show changes in the strength of compressive force across the whole length of a single tooth. Interfaces between each tissue with different individual structure and composition deflect the force from above without leading to catastrophic failure of the materials involved (Reproduced with kind permission from Elsevier with new labels added) [179].

## 3. Limitations and Challenges

The tooth organ is made up of an intricate assembly of hard and soft tissues. The softer part is often intercalated within the hardened mineralized tissues. Dentine, enamel, periodontal ligament and bone are structurally dissimilar but are integrated very well with each other. These components are held together with graded interlocking surfaces either with use of embedded fibers or by subtle melding of not-so dissimilar materials inside junctional zones such as the cementum-dentine junction (Figure 10) [179]. At the molecular level, special amphiphilic molecules have been adapted to bond two different materials together, usually through an intermediary organic phase. This organization transforms into vastly increased strengths at the macroscale. This level of sophistication has been hard to match at the level of detail adapted by evolution in living organisms. Such materials will likely have to be grown by cells rather than manufactured in the laboratory. Therefore, it is up to tissue engineering to craft environments and to potentiate the right assemblage of cellular building blocks.

Over the years, the pursuit of tooth regeneration and total tooth repair has been one of the main goals of research in regenerative and restorative dentistry. Since the turn of the last century, different biomaterials, including those from marine sources, have emerged as favorable candidates for tissue engineering and regeneration. Although we have discussed how these materials could facilitate tooth repair and regeneration in practical applications, materials and structures built from them do not meet all ideal criteria (including biomechanical, bioresponsiveness, ease of processing and storaging, cell and tissue growing efficiencies) needed for proper biological application (reviewed in Table 1). Marine biostructures are meeting these demands and have other significant advantages over existing biomaterials (Table 1). For example, marine biostructures usually have a hierarchy of built-in. Nevertheless, it is anticipated that with continuous advances in biochemical engineering and the continuous technical advances in materials modification to fine-tune material properties, a window of new opportunities will be opened for tooth regeneration and repair.

## 4. Conclusions

**Figure 11:**
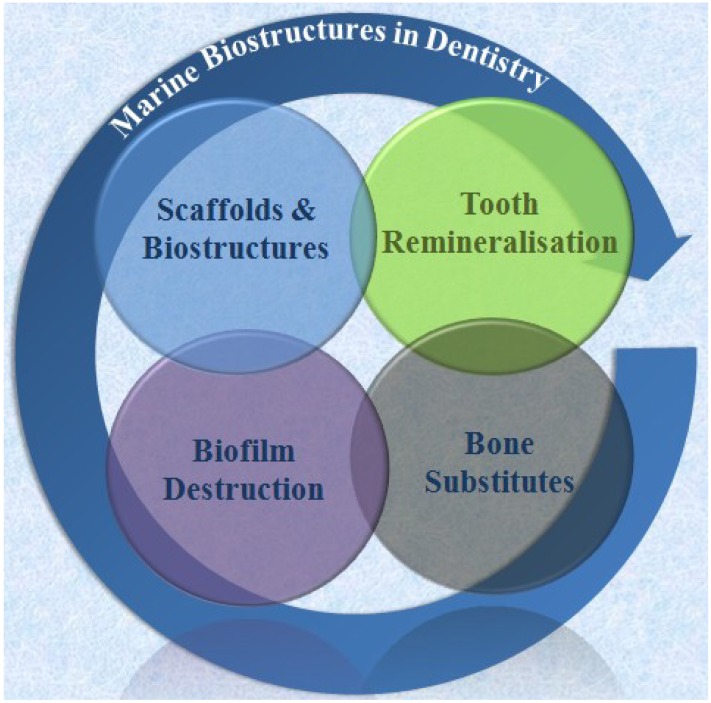
Major application areas for marine biostructures in modern dentistry that cover engineering of entire dental tissues and tissue complexes, disintegration of biofilms, repeat mineralisation of the tooth surface-enamel and shallow dentine defects- and bone substitutes.

We have demonstrated how a small selection of structural items from the marine environment can be usefully implemented in regenerative strategies in dentistry to help address common clinical problems, particularly those regarding loss of bone and dentine. These structural products range from small microscopic skeletons to large macroscopic structures. The different applications of marine products in dental tissue engineering have been summarized in Figure 11. Indeed, in addition to their use in dentistry, numerous materials derived from marine sources have been applied in medicine. Thus far, we have appropriated whole structures to create engineered bone, dentine and periodontal ligament tissues. These options are temporary and simple but exhibit potentially good tissue-producing outcomes. The objective is to establish the right environment and conditions for the onset of complete regeneration via a combination of exogenous and endogenous providers. Replicating dentine remains a challenge because the tissues that can be generated produce a reparative style of structure and not the true primary dentine. The periodontal ligament is a more significant challenge because graded joints are necessary on either side for anchorage to the bone and via the cementum layer onto dentine. The entire periodontium complex is an elaborate piece of graded engineering that has not been faithfully emulated artificially, biomimetically or via endogenous routes of action. The point of using natural structures in tissue engineering is to help build competent structural biomaterials that are the same as their natural archetypes or function in a manner that simulates the archetype (jawbone, dentine and periodontium). In these instances, the corals are a faithful mirror image of alveolar spongy bone, whereas the diatom spheres are biomimetic principally in function, striving to provide the right growth factor environment for faithful tissue formation. We have deliberately not looked at the wealth of materials and chemical compounds that could and have served purposes in engineering partial tooth structures. For example, alginates and chitosan have been crafted to engineer pulp tissue replacement with dental stem cells. Owing to the amazing diversity of marine life, there will be more significant discoveries in marine materials and biostructures for future implementation in regenerative dentistry. We envisage this approach to be a bio-based bridge from traditional acellular and non-biological dental biomaterials towards the final objective of a complete biomimetic tissue construct or whole tooth organ.
